# The Early Changes in Emergency General Surgery Following Implementation of UK COVID-19 Policy: A Retrospective Cohort Study

**DOI:** 10.7759/cureus.19832

**Published:** 2021-11-23

**Authors:** Joseph Hanger, Alexander Bush, Adam Lunt, Matthew Adams, Ben Keatley, Alicia Munro, Nasir Jaralla, Petros Christopoulos

**Affiliations:** 1 Gastrointestinal Surgery, Torbay and South Devon Hospital, Torquay, GBR

**Keywords:** operative, surgical procedures, public health, united kingdom government, general surgery, covid-19

## Abstract

Introduction

Coronavirus disease 2019 (COVID-19) has rapidly spread worldwide. On 23rd March 2020, the UK introduced measures in an effort to curb the disease spread. The aim of this study was to analyse the effect of government and Royal College measures on the general surgical take.

Materials and methods

A retrospective analysis of patients referred to the acute general surgical take between 2nd March 2020 and 5th April 2020, including acuity at the time of referral, management, and patient outcomes, was undertaken. Data fit into a ‘pre-COVID measures’ cohort (prior to 23rd March 2020) and a ‘post-COVID measures’ cohort (on or after 23rd March 2020).

Results

A total of 465 patient referrals were included. There was a decrease in admissions rate in the post-COVID measures’ cohort (p=0.001), but with an increase in patient acuity with white cell count (WCC) (p=0.024) and C-reactive protein (CRP) (p=0.036). Laparoscopic surgery decreased (p=0.004); however, the proportion of patients having an operation remained constant. There was no increase in short-term morbidity and mortality or length of stay (LOS).

Discussion

The data suggests that UK lockdown introduction influenced people’s behaviour. Fewer patients presented to the surgical take; however, these patients were of higher acuity. Despite changes in royal college guidelines, there was no decrease in the proportion of patients undergoing operations; however, a higher proportion were open procedures. The change in national and college guidelines did not affect short-term morbidity, mortality or LOS.

## Introduction

The highly infectious novel coronavirus disease COVID-19 is caused by severe acute respiratory syndrome coronavirus-2 (SARS-CoV-2). The virus was first identified in Wuhan province in China and a “pneumonia of unknown cause” was reported to the WHO on the 31st of December 2019 [1], which was later to be identified as and named COVID-19. Since then the infection has spread quickly across the globe. Governments around the world have responded to this by introducing measures to limit social contact to limit disease spread, and to increase the health care system’s ability to meet an expected increased demand for acute medical and intensive care beds. 

The first case of COVID-19 in the UK was reported on the 31st of January 2020. The government launched a public information campaign across the UK on 3rd February 2020, followed by its first policy paper on COVID-19 on the 3rd March 2020 which broadly outlined the strategy of contain, research, delay and mitigate [2]. 

The first informal measures to encourage social distancing however were put in place on the 16th March 2020 when the UK government issued advice to avoid venues such as pubs and restaurants. Increasingly strict measures followed over the course of the next few days. The first cases in our region were detected on the 2nd March 2020 and all elective operating in our hospital was stopped from the 16th of March (excepting urgent cancer surgery). The Surgical Royal Colleges of the UK published initial guidance on the 20th March 2020 which contained recommendations on how to fulfil the aims of maintaining emergency surgery capability, protect the workforce and fulfil alternative roles. This contained advice on altering our approach towards the management of certain surgical conditions, with the overarching principles of - “To triage and deliver healthcare to patients for maximal benefit as in a mass casualty scenario and to protect and preserve the surgical workforce” [3]. Initial intercollegiate general surgical guidance was split into five categories [4]: emergency surgery, planned surgery, theatre, laparoscopy, and endoscopy.

On 23rd March 2020, the Prime Minister addressed the nation introducing the first steps of a UK-wide lockdown, where leaving the house was restricted to meeting at least one of four criteria [5]; shopping for basic necessities, as infrequently as possible, one form of exercise a day - for example a run, walk, or cycle - alone or with members of your household, any medical need, to provide care or to help a vulnerable person, and travelling to and from work, but only where this is absolutely necessary and cannot be done from home.

Following the Government measures introduced on the 23rd March 2020, we anecdotally observed a reduction in the number of patients referred to the acute surgical take and an increase in the acuity of these patients, as well as some changes to their in-patient management. In this study, we evaluate those observations to determine if there is any objective evidence of these changes.

This article was previously been presented as a meeting abstract at the Association of Great Britain & Ireland (ASGBI) - Future Surgery ASGBI Virtual Congress on 7th May 2021.

## Materials and methods

A retrospective database of all patients presenting to the general surgical take between 2nd March 2020 and 5th April 2020 inclusive was accessed and analysis was conducted retrospectively. All patients who were referred for assessment or review by the on-call general surgical take team were included; this therefore included acute urology referrals. Elective patients were excluded. Collected data included patient demographics, referrer, presenting complaint, EWS at referral, admission blood tests, radiological tests, antibiotic use and operative and outcome data. Whether a COVID-19 test was performed during admission was also recorded.

Patients were then placed into either a ‘pre-COVID measures’ cohort (presenting prior to the 23rd March 2020) or a ‘post-COVID measures’ cohort (presenting on or after 23rd March 2020) and comparisons were made between the two. Primary outcome measures were number of referrals per day, acuity at the time of admission (as assessed by EWS, CRP, WCC and lactate on admission), morbidity and mortality. These factors are all predictors of morbidity and mortality [6-8]. Secondary outcome measures were radiological investigations performed, treatment with antibiotics, operations performed (and proportion which were laparoscopic) re-admission rate, proportion of patients ambulated, readmission rate and length of stay (LOS).

The objectives of the study and their outcome measures are summarised in Table 1.

**Table 1:** Summary of objectives and outcome measures. EWS: early warning score; WCC: white cell count; CRP: C-reactive protein; LFT: liver function test ; CXR: chest X-ray; AXR: abdominal X-ray; CT: computed tomography; MRI: magnetic resonance imaging.

Objectives	Outcome measures
Primary: Assess change in patient number and acuity of patients on admission to hospital	1. Number of referrals per day
	2. Patient acuity at admission (EWS, WCC, CRP, LFTs amylase, lactate)
Secondary: Assess differences in patient investigation, management and outcomes	2.1 Radiological investigations (CXR, AXR, CT, MRI)
	2.2 Clinical management (antibiotic therapy, intensive care, length of stay, ambulatory care)
	2.3 Operative management (type, laparoscopy, primary surgeon)
	2.4 Morbidity and mortality

Morbidity was defined and categorised as per the Clavien-Dindo index, and divided into minor (Clavien-Dindo index 1-2), or major (Clavien-Dindo index 3-4) and Death (Clavien-Dindo index 5). Each patient’s most significant complication was recorded as their morbidity score, as per the index [9].

Descriptive statistics were used for categorical data and Shapiro-Wilkes testing of normality for continuous data. The study assumed that P values of less than 0.05 were significant.

The study was registered online Researchregistry.com (Researchregistry5786). Based on local policies ethical approval and individual informed consent was not required for this study. The study was reported in line with the STROCSS criteria [10].

## Results

In total 465 patients were referred to the general surgical take during the study period. Baseline characteristics are summarised in Table 2. There was no significant difference in mean age at presentation, ratio of male:female patients, referral source or presenting complaint.

**Table 2:** Baseline characteristics. ED: emergency department; GP: general practice/practitioner; GI: gastrointestinal.

	Pre-measures cohort	Post-measures cohort	p-value
N=	331	132	0.0013
Mean age (years)	54.72	51.41	0.1827
Sex (n)			0.5401
Male	166	62	
Female	165	71	
Referral source (n)			0.7383
ED	192	78	
GP	98	41	
Other	41	13	
Presenting complaint			0.6229
General surgery	192	79	
Colorectal	41	11	
Upper GI	32	17	
Urology	40	14	
Other	27	12	

Referral rate

**Figure 1:**
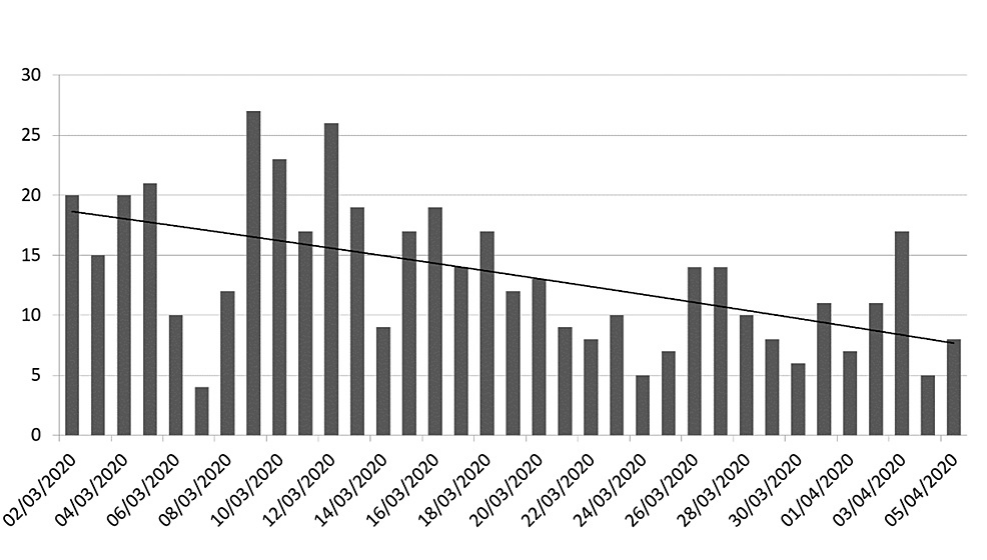
Histogram to demonstrate trend in the number of surgical referrals per day (linear).

There was a gradual reduction in the admission rate over the study period (Figure 1). When patients were evaluated as cohorts there was a significant reduction in the number of surgical reviews per day in the post-measures cohort compared with the pre-measures cohort (M=9.5, SD=1.0 vs M=15.8, SD=2.6, p=0.001).

Acuity at admission

There was a significant increase in mean CRP (39.4 to 52.6, p=0.04), WCC (10.7 to 12.2, p=0.02) and amylase (p=0.008) at presentation in the post-measures cohort (Table 3). There was, however, no significant increase in lactate or EWS at the point of referral. Not all patients received all blood tests.

**Table 3:** Mean blood test results for patients at the point of referral. EWS: early warning score; WCC: white cell count; CRP: C-reactive protein.

	Pre-measures cohort (±SD)	Post-measures cohort (±SD)	Difference	p-value	
					Mean CRP (n=438)	39.4 ± 65	52.6 ± 81	13.2	0.036	
Mean WCC (n=446)	10.7 ± 4.8	12.2 ± 9	1.5	0.024	
Amylase (n=255)	67.6 ± 82.4	175.8 ± 536	108.2	0.008	
EWS (n=465)	1.5 ± 2	1.5 ± 1.8	0.0	0.947	
Lactate (n=285)	1.85 ± 1.4	1.86 ± 1.8	0.01	0.681	

Imaging

Imaging practices were compared pre- and post-measures being put in place (Table 4). There was a significant change in the proportion of chest radiographs being completed during admission (40.6% compared with 36.8%, p=0.048). There were no other significant differences in imaging practices.

**Table 4:** Proportion of patients receiving an imaging modality during their admission. Percentage is given as percentage of cohort (with Chi-squared analysis). CT: computed tomography; MRI: magnetic resonance imaging.

	Pre-measures cohort (n=332)	Post-measures cohort (n=133)	p-value
Chest radiograph	122 (36.8%)	54 (40.6%)	0.048
Abdominal radiograph	92 (27.7%)	42 (31.6%)	0.41
CT	104 (37.6%)	39 (43.0%)	0.67
Ultrasound	73 (22.0%)	36 (27.1%)	0.24
MRI	5 (1.5%)	3 (2.3%)	0.57

Management

There was no significant change in antibiotic usage during the study (47.59% vs 41.35%, p=0.223). There was a reduction in the proportion of patients admitted to ICU in the post-measures cohort although this did not reach statistical significance (3.31% vs 1.50%, p=0.285). 

**Figure 2:**
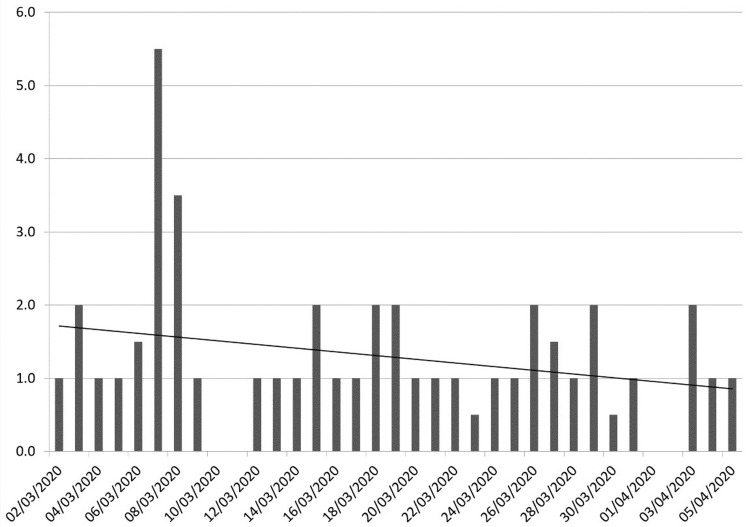
Trend in median length of stay in days (linear).

There was a general downward trend in the length of stay during the study period (Figure 2). This was likely multifactorial and an avenue to future research based on previous years data.

The median LOS in both cohorts, however, was one day (p=0.652). There was also no significant difference in the proportion of patients treated using an ambulatory pathway. (i.e. being assessed in hospital and discharged home on the same day to return for further investigation or treatment) (p=0.642) (Table 5).

**Table 5:** Inpatient management.

	Pre-measures cohort (n=332)	Post-measures cohort (n=133)	p-value
Antibiotic usage	158 (47.59%)	55 (41.35%)	0.224
Length of stay (median)	1	1	0.652
Ambulatory pathway	66 (19.88%)	29 (21.89%)	0.642
COVID-19 test	6 (1.8%)	27 (20.3%)	0.00001

There was a significant increase in the proportion of patients having COVID-19 tests (p<0.00001). One patient tested positive for the virus during the study period. There was no significant difference in the proportion of patients undergoing operative management (26.3% vs 22.6%, p=0.413). There was a significant decline in laparoscopic surgery in the post-measures cohort (p=0.004) and an increase in hernia repairs over the same period (p=0.015). Otherwise, there was no significant change in the types of operations performed (Table 6).

**Table 6:** Number and types of operations performed. EUA: examination under anaesthesia.

	Pre-measures cohort	Post-measures cohort	p-value
	(n=87)	(n=30)	
Laparoscopic surgery	30	2	0.004
Cholecystectomy	8	0	0.111
Appendicectomy	17	7	0.79
Incision & drainage	20	7	1
Small bowel resection	2	2	0.271
Division of adhesions	3	2	0.602
Hernia repair	1	4	0.015
EUA rectum	3	1	1
Hartmann’s	2	0	1
Colostomy	1	0	1
Ureteric stent	1	1	0.449
Flatus tube	1	1	0.449
Delormes	1	0	1
Other	1	3	0.051

Morbidity and mortality

Comparison of morbidity and mortality shows there was no significant change in overall morbidity (p=0.861), minor (p=0.998) or major (p=1) complications, inpatient mortality (p=0.680) or 30-day mortality (p=0.940) (Table 7). Overall reattendance rate was 9.8%, with no significant difference between the pre and post-measures cohorts (p=0.0631). 

**Table 7:** Morbidity and mortality as proportion of patients seen.

	Pre-measures cohort	Post-measures cohort	p-value
	(n=332)	(n=133)	
Inpatient morbidity	6.30%	6.80%	0.861
Clavien-Dindo Minor (1-2)	3.00%	3.00%	0.997
Clavien-Dindo Major (3-4)	0.30%	0%	1
Inpatient mortality	3.00%	3.80%	0.68
30-day mortality	3.75%	3.80%	0.94
Reattendance rate	10.20%	5.30%	0.0631

## Discussion

The COVID-19 global pandemic has led to governments around the globe introducing a policy to reduce infection spread. Surgical societies have also produced guidance with the aim of maintaining emergency surgery capacity whilst protecting both the workforce and the general population. This study has specifically evaluated the effects of what have become known as the ‘lockdown’ measures introduced on the 23rd March 2020. This study has been conducted in a district general hospital, in an area of the country where some of the first UK cases of COVID-19 were identified.

Due to the novelty of this virus, there is little published data about the effects of the pandemic, and the measures taken to fight it on emergency surgery services. There have been several reviews looking how best to continue with safe surgical practice during the pandemic [11] and a small retrospective study (n=34) from Wuhan showed high mortality (20.5%) in elective surgical patients who had COVID-19 at the time of operation [12]. This is supported by the COVIDSurg collaborative multi-centre study showing similar 30-day mortality (23.8%) [13].

Our study, evaluating 465 patient referrals, has shown a reduction in daily referrals to the on-call surgical team (p=0.001). This was in conjunction with an increased acuity with both a rise in CRP and WCC at the point of referral. These changes are likely multifactorial. Patients with more minor complaints are likely to have avoided attending hospitals in response to calls to protect the NHS. Colleagues in primary care sought ways to manage patients without hospital admission in order to reduce pressures on hospitals and to maintain hospital capacity. Other patients were reluctant to attend healthcare facilities due to fears over contracting COVID-19 and therefore delayed their presentation. In the longer term it will be important to study patients who did not present to the general surgical take as a result of COVID-19 both qualitatively and quantitatively to better understand these observed changes and if possible to measure any resultant effects on their health.

COVID-19 is a medical respiratory illness with classical respiratory findings seen on both X-ray and CT [14]. There was a significant increase in the number of chest radiographs performed following the introduction of lockdown measures. This change was likely due to a reduction in the threshold to perform a chest x-ray for patients presenting with symptoms that could be considered COVID-19 related. It was recognised early in the pandemic that chest CT is a sensitive test to identify COVID-19 with evidence of infection on CT found in 97% of those with a positive PCR test [3]. There was also guidance that recommended conservative approaches opposed to surgical management when possible. We therefore hypothesised that there would be a significant increase in the proportion of patients undergoing CT to help guide conservative management. Our study showed an increase in patients having a CT from 37-43% however this did not reach significance(p=0.67). A possible reason for this finding could be some patients presenting later in their disease process and there was less diagnostic uncertainty, reducing the need for cross-sectional imaging. 

Laparoscopic surgery is considered an aerosol-generating procedure as release of the pneumoperitoneum upon removing ports can release particles under pressure. The presence of COVID-19 in the peritoneal fluid has been reported [3]. A significant guideline regarding operative management of patients during the COVID-19 pandemic was that laparoscopic surgery should be avoided if at all possible and considered only in selected individual cases where the clinical benefit to the patient substantially exceeds the risk of potential viral transmission in that particular situation [3]. In our study, there was a significant decrease in the proportion of operations that were laparoscopic (p=0.004) suggesting good compliance with this guidance. A further systematic review around the effectiveness of this guideline has shown no evidence for the transmission of COVID-19 by laparoscopic surgery [15]. Although does continues to support the use of laparoscopic surgery with precautions throughout the pandemic.

The proportion of patients having operations did not change significantly during the study period. A statistically significant difference was noted in the number of hernia repairs performed however the data regarding case mix is difficult to analyse due to the small sample sizes. The results of the larger collaborative studies such as CovidSurg will provide more information on operative practices and outcomes.

Despite the increase in acuity at presentation and increased use of open surgery instead of laparoscopic techniques, there was reassuringly no significant overall morbidity (p=0.861), minor (p=0.998) or major (p=1) complications, inpatient mortality (p=0.680) 30-day mortality (p=0.940) or LOS.

This study has some limitations, notably it was a single-centre study of a district general hospital. It presents data and conclusions drawn from only a single unit at the beginning of the COVID-19 pandemic, from a single region within the UK. Further much larger multi-centre, but also more importantly multi-regional study would give a much better view of the effects of emergency general surgical. A further limitation of the study is that mortality and morbidity data were collected short-term. The full effects of COVID on the morbidity and mortality of patients are likely to be long-standing and may not be fully recognised for several years. Further studies should consider looking at long-term morbidity and mortality to emergency general surgical patients who have presented to the general surgical take during the COVID-19 pandemic. 

This study puts forward a single-centre experiences during the first wave of the COVID-19 pandemic, going forward it will add to the body of research helping support policymakers, clinicians and hospitals plan for the care of emergency general surgical patients during future waves of the COVID-19 pandemic as well as other global pandemics. 

## Conclusions

In conclusion, this study demonstrates that COVID-19 and the measures put in place to tackle it have had a significant effect on the general surgical take in our unit by decreasing referral numbers and changing the management of patients. Despite an increase in the acuity of patients at presentation and changes to both their inpatient and operative management, there has been no significant short-term effect on overall outcomes for those patients who have been referred to the general surgical take.
